# Intestinal Intussusception Secondary to Meckel's Diverticulum: A Case Report

**DOI:** 10.7759/cureus.82730

**Published:** 2025-04-21

**Authors:** Christian Ballardo Medina, Jose Manuel Rocha Chavez, Maria V Figueroa Beltran, Paul Humberto Valdez Castillejo, Rodolfo Lopez Hernandez, Ana Karen Carrasco Gaxiola

**Affiliations:** 1 General Surgery, Instituto de Seguridad Social para los Servidores Públicos del Estado, Autonomous University of Sinaloa, Culiacán, MEX

**Keywords:** acute abdomen in children, emergency surgical care, explorative laparotomy, ileo-ileal intussusception, meckel´s diverticulum

## Abstract

Meckel's diverticulum (MD) is the most common small intestine anomaly. Although it is often asymptomatic, in pediatric patients it typically presents as obstruction or lower gastrointestinal bleeding due to ileo-ileal intussusception. We present the case of a pediatric patient in his second decade of life with symptoms of an acute abdomen and laboratory abnormalities suggesting an infectious or inflammatory process. A computed tomography scan revealed small bowel dilation, increased bowel wall diameter, and air-fluid levels. He underwent exploratory laparotomy, which revealed an intussusception of a MD. The affected segment was resected, followed by an end-to-end anastomosis. In cases where complications of MD are strongly suspected, the surgeon should opt for surgical management as soon as possible, whether by laparotomy, laparoscopy, or laparoscopically assisted surgery. Regardless of the available options, an appropriate decision should be made based on intraoperative findings and potential postoperative complications. Surgical treatment should depend on intraoperative findings and the surgeon's expertise to ensure a safe and complication-free procedure.

## Introduction

Meckel's diverticulum (MD) is defined as the persistence of the omphalomesenteric duct and is the most common small intestine anomaly, characterized as a true diverticulum [1]. A well-known characteristic of MD is the "rule of 2s": it is located approximately 2 feet from the ileocecal valve, measures more than 2 inches in length, has a 2:1 male-to-female ratio, occurs in 2% of the population, is symptomatic in only 2% of cases, and commonly contains two types of ectopic mucosa: gastric and pancreatic [2,3]. While MD is often asymptomatic, pediatric patients frequently present with obstruction or lower gastrointestinal bleeding due to ileo-ileal intussusception. In contrast, in adults, it occurs in approximately 1 in every 300-400 acute abdomen cases, with some authors reporting it in 4% of all intussusception cases [4,5].

## Case presentation

A 14-year-old male presented with sudden, persistent abdominal pain, predominantly in the right hypochondrium, rated 7/10 on the pain analog scale, lasting 12 hours without relief from positional changes. The pain was accompanied by nausea and vomiting of gastrointestinal characteristics. The patient had no relevant medical, surgical, or allergic history. Laboratory tests showed leukocytosis (12.64 × 10³/mm³; reference value (RV): 4.5-12 × 10³/mm³) with neutrophilia (86.9%; RV: 37-75%), prolonged prothrombin time (15.6 seconds; RV: 11.5-15.5 seconds), and negative procalcitonin (0.0 ng/dL; RV: 0-0.5 ng/dL).

**Figure 1:**
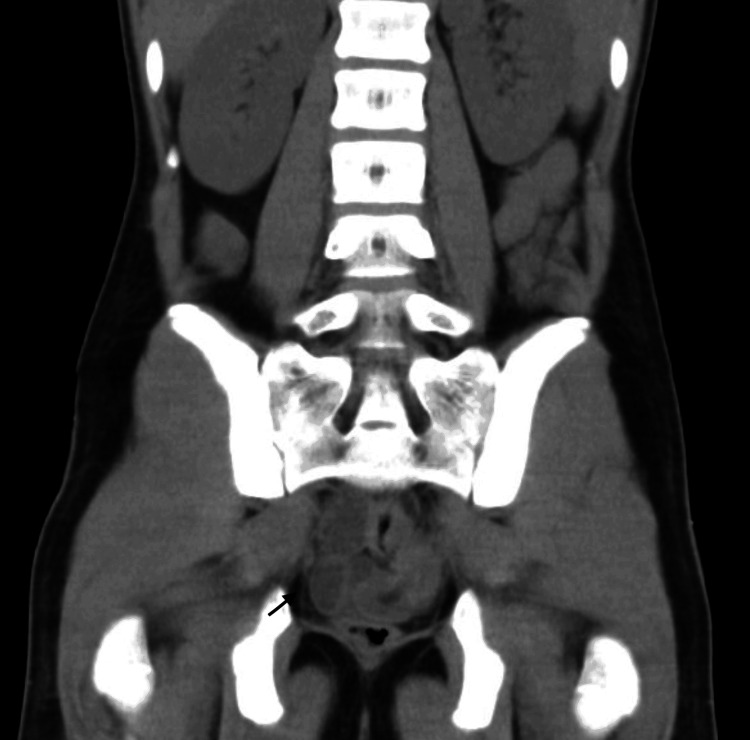
Coronal CT scan showing a longitudinal segment of intussusception with thickened walls and fatty content within the colon segment.

**Figure 2:**
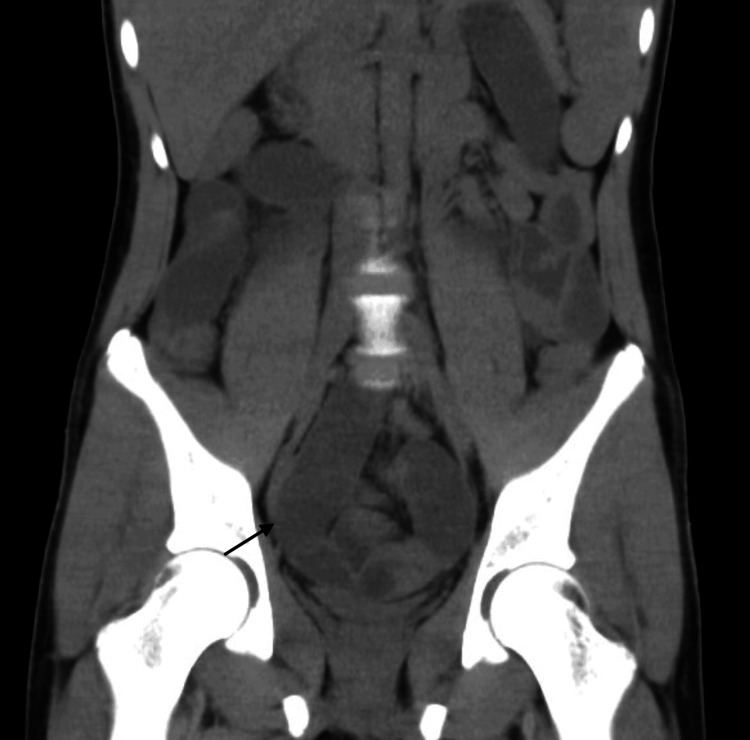
Dilated ileal segment preceding the intussusception.

A non-contrast abdominopelvic CT scan revealed dilated intestinal loops up to 30 mm in diameter with air-fluid levels and minimal air in the rectal ampulla, suggestive of generalized ileus or a progressive obstructive process (Figures 1, 2).

**Figure 3:**
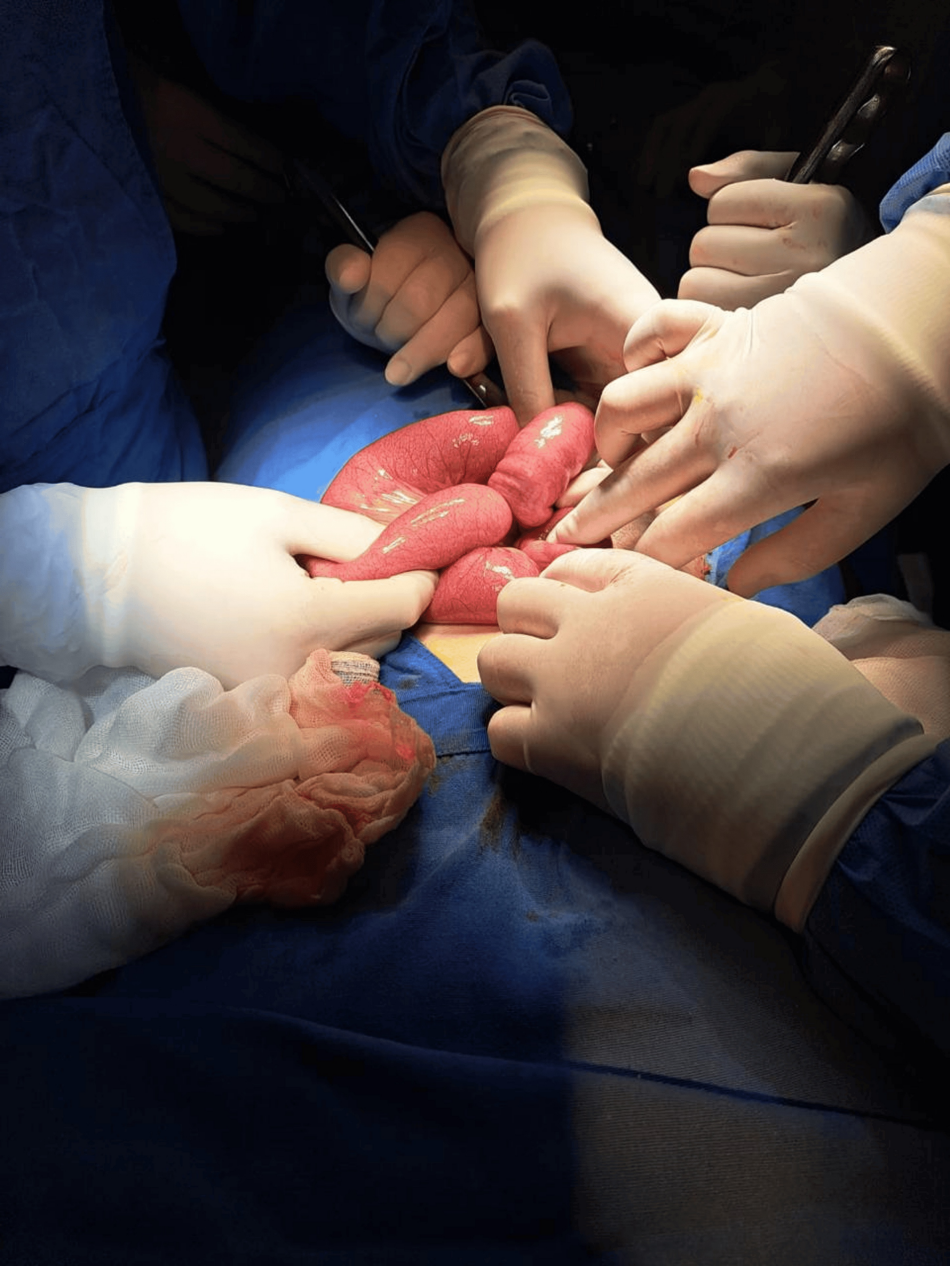
Intussusception of Meckel's diverticulum.

**Figure 4:**
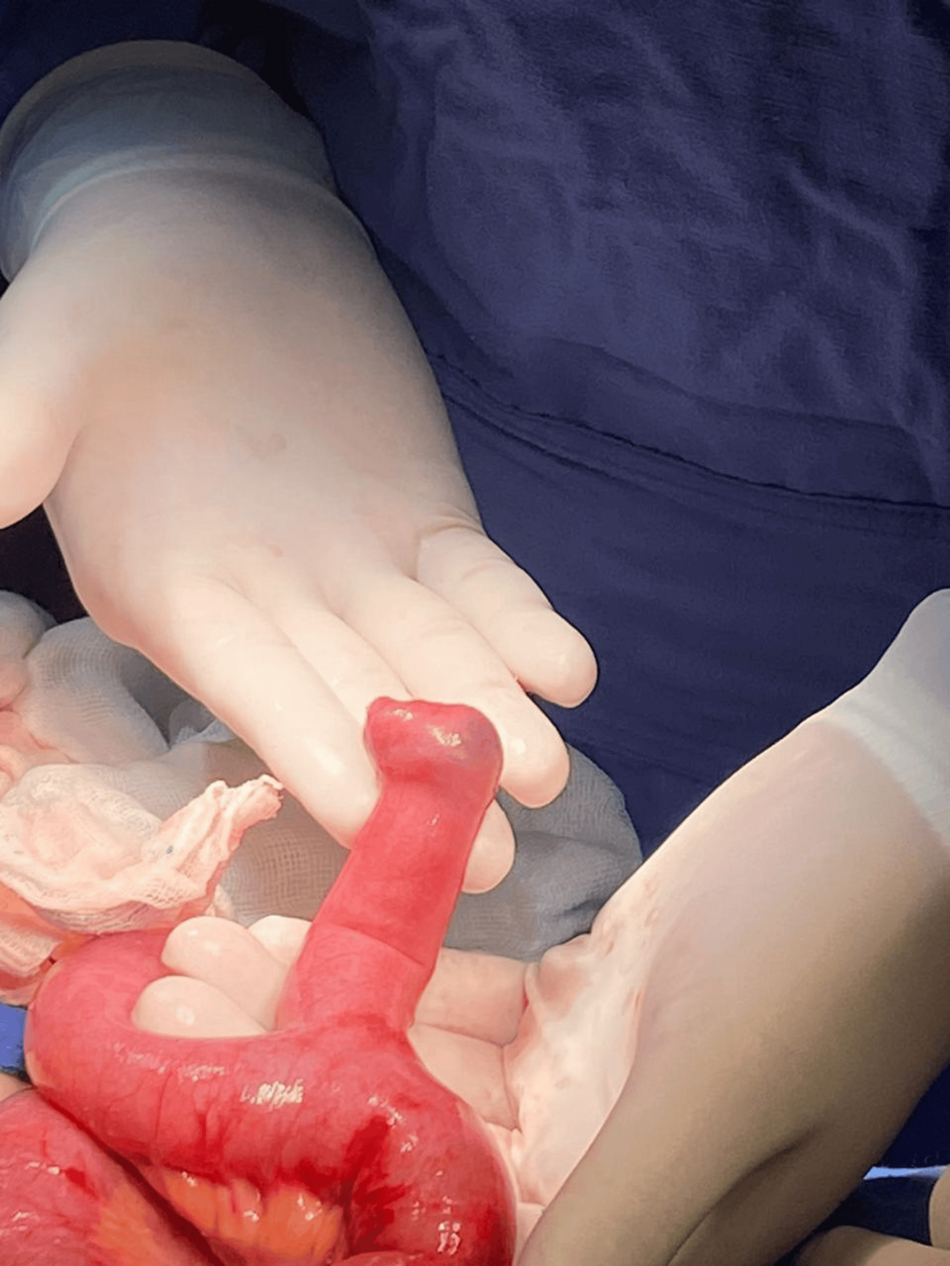
Isolated Meckel’s diverticulum, measuring approximately 10x3 cm.

The patient underwent exploratory laparotomy, which revealed intussusception 60 cm from the ileocecal valve. Manual reduction was performed, identifying an MD measuring approximately 10 × 3 cm (Figures 3, 4). A wedge resection of the ileum, including the diverticulum, was performed with a 5 cm margin proximally and distally, followed by end-to-end anastomosis using Cushing's suture pattern with 3-0 Vicryl and reinforcement with Lembert's suture pattern using 2-0 silk. The procedure was completed without complications.

Postoperatively, the patient tolerated oral liquids 12 hours after surgery and ambulated without significant pain. A soft diet was introduced on postoperative day 3, and the patient was discharged on postoperative day 5. Pathological analysis confirmed an MD measuring 5.8 × 1.5 cm with ectopic gastric tissue.

## Discussion

MD presents with diverse clinical manifestations and is often asymptomatic. Complications include bleeding, intestinal obstruction, ulceration, hemorrhage, and perforation, with intussusception being a very rare presentation [6].

A high degree of suspicion and imaging studies are crucial for diagnosing intussusception caused by MD, given its insidious presentation. Available imaging modalities range from simple radiographs to nuclear imaging. Plain X-rays and CT scans provide limited support, often revealing signs of intestinal obstruction and, in later stages, intestinal perforation. In pediatric patients, ultrasonography is recommended, as it can show tubular, blind-ended loops, segmental thickening, or pelvic abscesses. Advanced imaging modalities such as superior mesenteric artery angiography, technetium-99 nuclear scans, capsule endoscopy, double-balloon enteroscopy, or CT/MRI enterography are more sensitive but may not be readily available in emergency settings [7]. In this case, an abdominopelvic CT scan showed thickening and distension of the distal ileal walls.

When MD complications are suspected, surgical intervention should be performed promptly via laparotomy, laparoscopy, or laparoscopically assisted surgery. Regardless of surgical limitations, an appropriate decision must be made based on intraoperative findings and potential postoperative complications. Treatment options include diverticulectomy, particularly in elective cases or incidental findings during appendectomy, and segmental intestinal resection with anastomosis, especially when preoperative studies indicate palpable ectopic tissue at the diverticular-intestinal junction, intestinal ischemia, or perforation [8-10]. In this case, the patient presented with an acute abdomen and underwent exploratory laparotomy, revealing intussusception due to MD. A wedge resection was performed, followed by an end-to-end isoperistaltic anastomosis, resulting in a favorable postoperative course.

## Conclusions

Intussusception caused by MD is a rare condition, typically presenting as intestinal obstruction. Surgeons should maintain a high index of suspicion for early diagnosis, with surgical treatment being the cornerstone of management. The choice of surgical approach should be based on intraoperative findings and the surgeon's expertise to ensure a safe and complication-free procedure. Further analysis and studies are required to determine predictive factors for complex pathology, which would allow for less invasive procedures.
